# Toward a unified understanding of drug-drug interactions: mapping Japanese drug codes to RxNorm concepts

**DOI:** 10.1093/jamia/ocae094

**Published:** 2024-05-17

**Authors:** Yukinobu Kawakami, Takuya Matsuda, Noriaki Hidaka, Mamoru Tanaka, Eizen Kimura

**Affiliations:** Department of Medical Informatics, Ehime University Graduate School of Medicine, Shitsukawa, Toon, Ehime, 791-0295, Japan; Division of Pharmacy, Ehime University Hospital, Shitsukawa, Toon, Ehime, 791-0295, Japan; Department of Medical Informatics, Ehime University Graduate School of Medicine, Shitsukawa, Toon, Ehime, 791-0295, Japan; Division of Pharmacy, Ehime University Hospital, Shitsukawa, Toon, Ehime, 791-0295, Japan; Division of Pharmacy, Ehime University Hospital, Shitsukawa, Toon, Ehime, 791-0295, Japan; Department of Medical Informatics, Ehime University Graduate School of Medicine, Shitsukawa, Toon, Ehime, 791-0295, Japan

**Keywords:** drug codes, drug-drug interaction, knowledge base, RxNorm, terminology

## Abstract

**Objectives:**

Linking information on Japanese pharmaceutical products to global knowledge bases (KBs) would enhance international collaborative research and yield valuable insights. However, public access to mappings of Japanese pharmaceutical products that use international controlled vocabularies remains limited. This study mapped YJ codes to RxNorm ingredient classes, providing new insights by comparing Japanese and international drug-drug interaction (DDI) information using a case study methodology.

**Materials and Methods:**

Tables linking YJ codes to RxNorm concepts were created using the application programming interfaces of the Kyoto Encyclopedia of Genes and Genomes and the National Library of Medicine. A comparative analysis of Japanese and international DDI information was thus performed by linking to an international DDI KB.

**Results:**

There was limited agreement between the Japanese and international DDI severity classifications. Cross-tabulation of Japanese and international DDIs by severity showed that 213 combinations classified as serious DDIs by an international KB were missing from the Japanese DDI information.

**Discussion:**

It is desirable that efforts be undertaken to standardize international criteria for DDIs to ensure consistency in the classification of their severity.

**Conclusion:**

The classification of DDI severity remains highly variable. It is imperative to augment the repository of critical DDI information, which would revalidate the utility of fostering collaborations with global KBs.

## Introduction

**Figure 1.**
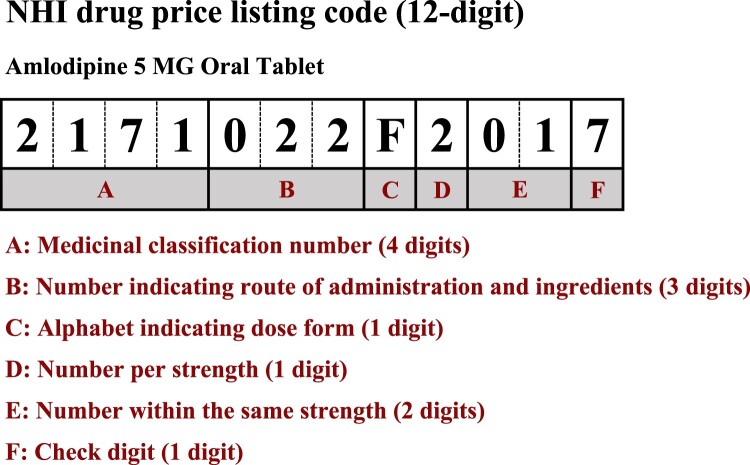
The National Health Insurance (NHI) drug price listing code.

Recently, the mapping of various pharmaceutical terms to internationally controlled vocabularies has facilitated the global sharing of medical and drug information and enhanced boundary-free clinical research.^1–4^ Although few public mappings of Japanese pharmaceutical products employ these vocabularies, their use could promote international collaborative research and yield new knowledge. Japanese pharmaceutical products are encoded in various formats for different purposes. The major drug codes are a National Health Insurance (NHI) drug price listing code and an individual drug code (the “YJ code”), both of which are commercial product codes.^5^ The NHI drug price listing code assigns a unique 12-digit alphanumeric identifier to each drug price in a process controlled by the Japanese Ministry of Health, Labour and Welfare (Figure 1). The NHI drug price system defines drugs, the costs of which are reimbursed, and the prices. Both “Brand Name Listing” (BNL) and “Unified Name Listing” (UNL) are used and mixed in the same list. BNL assigns a unique code to each brand of drug, whereas UNL allocates a single code to multiple drugs with the same ingredients, dose forms, and strengths. Thus, the UNL does not uniquely identify a specific drug. BNL consists of ingredients, dose forms, strengths, and brand names, while UNL consists of ingredients, dose forms, and strengths.

**Figure 2.**
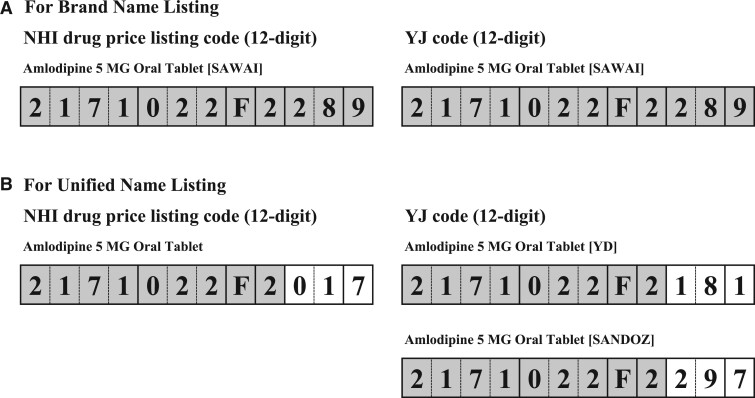
Relationship between the National Health Insurance drug price listing code and the YJ code.

The YJ code, also a 12-digit alphanumeric code, is similar to BNL and consists of ingredients, dose forms, strengths, and brand names. We selected the YJ code as the mapping target in this study because, unlike the NHI drug price listing code, it assigns separate codes to each drug (Figure 2), to uniquely identify all drugs.

RxNorm is a terminology component of the Unified Medical Language System,^6^ which facilitates mappings with other controlled vocabularies.^7^ The Observational Health Data Sciences and Informatics (OHDSI) project, which is operated by an international real-world data research group,^8^ developed the OHDSI Standard Vocabulary, which integrates and facilitates analyses of country-specific real-world data by mapping national pharmaceutical terms to RxNorm concepts. Thus, RxNorm acts as a bridge that facilitates knowledge acquisition and international clinical research; the various knowledge bases (KBs) are integrated. Yu et al^9^ showed that mapping pharmaceutical terms of the Food and Drug Administration (FDA) Adverse Event Reporting System (FAERS) to RxNorm concepts would facilitate integration with (eg) electronic health records and enhance the detection of adverse event signals. Thus, mapping concepts and linking medicinal data to KBs offer tangible clinical and research benefits. For this reason, mapping Japanese pharmaceutical products to RxNorm concepts should facilitate international clinical research in the OHDSI context and linkage to the KBs built by several other controlled vocabularies.

In this study, we linked the drug information databases of Japan and the United States and mapped Japanese pharmaceutical products to RxNorm concepts with respect to ingredient classes. Japanese and international drug-drug interactions (DDIs) were compared using ingredient classes as a case study approach; we sought to extract new knowledge that would contribute to progress in international research.

## Methods

### Correspondence between YJ codes and RxNorm concepts

To establish a connection between Japanese drug information and international KBs, the mapping targets were the YJ codes commonly utilized in Japan and RxNorm concepts widely employed in international research. Mapping data were extracted from the Kyoto Encyclopedia of Genes and Genomes (KEGG) MEDICUS database,^10^^,^^11^ which integrates a genome network (KEGG NETWORK), disease (KEGG DISEASE), and drug (KEGG DRUG) databases with drug labels in Japan and the United States. Drug labels are linked based on the drug ingredients. The KEGG DRUG database centrally manages pharmaceutical products in both Japan and the United States using unique KEGG D numbers.

**Figure 3.**
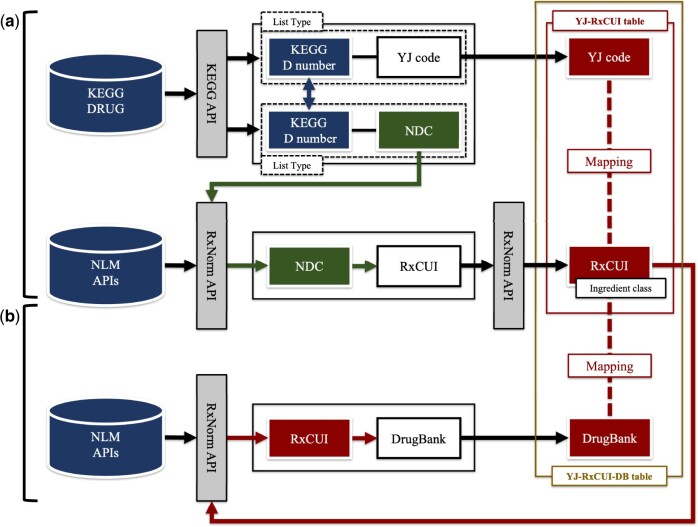
Mapping between controlled vocabularies.

We used Python v3.9.7 to access the KEGG application programming interface (API)^12^ and extracted tables of the YJ codes and the U.S. National Drug Codes (NDCs) linked to KEGG D numbers. Then, we used NLM RxNorm API^13^ to obtain RxNorm Concept Unique Identifiers (RxCUIs) corresponding to the NDCs. We excluded allergen extracts because the relevant Japanese and international items differed markedly. Finally, we prepared a correspondence (YJ-RxCUI) table linking the YJ codes to ingredient class-equivalent RxCUIs (Figure 3a); we manually verified the agreement of ingredient notations between the YJ codes and RxCUIs. Verification was conducted by a pharmacist with 5 years of professional experience.

### Correspondences among YJ codes, RxNorm concepts, and DrugBank identifiers

The DDI KB^14^ developed by Ayvaz et al was the international KB of choice; it manages drugs using DrugBank identifiers, or accession numbers.^15^ We used the RxNorm API to acquire DrugBank identifiers corresponding to the RxCUIs and prepared a correspondence table of YJ codes, RxCUIs, and DrugBank identifiers, that is, a YJ-RxCUI-DB table (Figure 3b). All oral and injectable drug ingredients listed by the Japanese NHI drug price were included.

### Comparison of Japanese and international DDI information

The API collected Japanese DDI information from the KEGG MEDICUS DDI database; it consists of information on Japanese drug labels, and the drugs are coded using the KEGG D numbers. We examined the correspondence between KEGG D numbers and the YJ codes of the KEGG DRUG database; this yielded DDI information corresponding to the YJ codes. The DDI severity classifications on Japanese drug labels are “Contraindications” and “Precautions.” The international DDI information was that of the KB of Ayvaz et al^14^^,^^16^ and consisted of an amalgamation of several DDI databases. The databases that yielded DDI severity information for the KB of Serkan et al included the FrenchDB,^17^ NDF-RT,^18^ OSCAR,^19^ World Vista,^20^ ONC High Priority,^21^ and ONC Noninterruptive.^22^ The data sources used in this study consisted of DDI information containing only ingredient classes. The DDI severity classifications are shown in Table 1.

**Table 1.** Drug-drug interaction (DDI) severity classifications.

Source	Severity	Description
FrenchDB	CI	Contraindicated
	AD	Avoid if possible
	PE	Take precautions when using; pharmacokinetic interactions may be in action
	PC	Consider possible pharmacodynamic interactions
NDF-RT	Critical	“Critical” for DDIs that the system's developers consider to be of greater concern than “Significant”
	Significant	
OSCAR	3	Significance increasing from 1 to 3
	2	
	1	
World Vista	Contraindication	Note the severity classification
	Not recommended	
	Precaution for use	
	Take into account	
ONC	High Priority	A list of DDIs that the Office of the National Coordinator considers should raise high-priority alerts
	Noninterruptive	A list of DDIs that the Office of the National Coordinator considers should be used for noninterruptive alerts
KEGG	CI	The KEGG DDI database contains known DDIs associated with contraindications (CI) and precautions (P)
	P	

Abbreviation: KEGG, Kyoto Encyclopedia of Genes and Genomes.

The FrenchDB, NDF-RT, OSCAR, and World Vista DDI severity classifications are unique. Conversely, the ONC High Priority and ONC Noninterruptive construct lists according to DDI severity and relevant data identify the severity through list affiliation. As shown in Table 1, it was impossible to align the DDI severity with a unified classification criterion because the granularity of DDI severity classification varies across sources. Therefore, we tentatively classified the DDI severity in this research to highlight how widely differing opinions on high severity could exist, even within the DDI classification criteria. We defined 3 levels: “Highest class,” “Other class” (less high), and “No information.” The controlled severity classifications and numbers of DDIs are shown in Table 2.

**Table 2.** Controlled severity classifications and numbers of DDIs.

Source	Severity	Severity term	Number of DDIs
FrenchDB	CI	Highest class	1820
	AD	Other class	60 227
	PE		
	PC		
NDF-RT	Critical	Highest class	688
	Significant	Other class	1188
OSCAR	3	Highest class	451
	2	Other class	8657
	1		
World Vista	Contraindication	Highest class	1527
	Not recommended	Other class	12 166
	Precaution for use		
	Take into account		
ONC	High Priority	Highest class	1930
	Noninterruptive	Other class	2101
KEGG	CI	Highest class	3230
	P	Other class	112 589

Abbreviations: DDIs, drug-drug interactions; KEGG, Kyoto Encyclopedia of Genes and Genomes.

**Figure 4.**
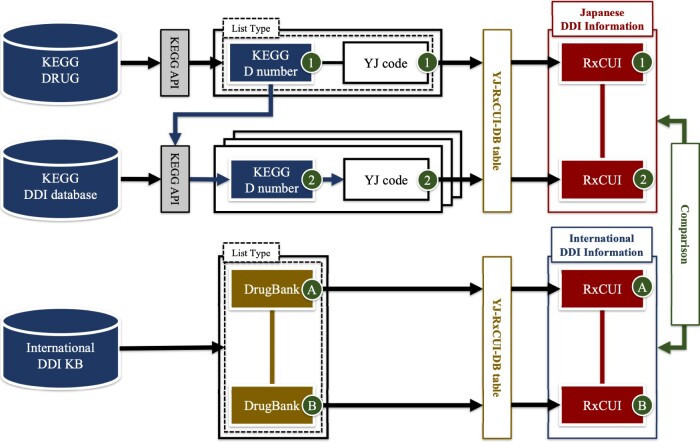
Comparison of Japanese and international drug-drug interaction information.

Then, we used the YJ-RxCUI-DB table to integrate the Japanese and international DDI information and compared the DDI severity classifications (Figure 4) using Cohen’s kappa values^23^ calculated by the sklearn.metrics module of the Python scikit-learn library v1.2.1. Visualization was aided by the heatmap function of the Python seaborn library v0.11.2. We used the Cohen’s kappa rating scale proposed by McHugh.^24^ Finally, all controlled severity classifications were cross-tabulated to compare Japanese and international DDI information.

## Results

### Correspondence between YJ codes and RxNorm concepts

A total of 49 192 drugs with corresponding YJ codes and NDCs were extracted from the KEGG DRUG database. The YJ-RxCUI table revealed 11 239 correspondences. Validation revealed that 10 823 (96%) were accurate, whereas 416 (4%) were not.

### Correspondences among YJ codes, RxNorm concepts, and DrugBank identifiers

A total of 1016 unique RxCUIs were listed in the YJ-RxCUI table. DrugBank identifiers were obtained for 981 (97%). Eleven RxCUIs exhibited a 1:2 correspondence between RxCUIs and DrugBank identifiers; in each case, one of the 2 was excluded to establish a 1:1 relationship after meticulously evaluating the correspondences. The remaining 35 RxCUIs (3%) that lacked correspondences were mapped manually using a DrugBank web-based search.^25^ Six were excluded due to the lack of DrugBank identifiers corresponding to the RxCUIs. The YJ-RxCUI-DB table included oral and injectable drug ingredients listed by the Japanese NHI drug price; there were 9242 correspondences.

### Comparison of Japanese and international DDI information

A total of 498 017 DDIs were extracted from the KEGG MEDICUS DDI database; after converting YJ codes to RxCUIs and excluding duplicates, including symmetric correspondences (eg, A-B, B-A), 31 865 DDIs remained. A total of 200 159 DDIs were extracted from the international KB; of these, 152 lacking DrugBank identifiers were mapped manually using a DrugBank web-based search.^25^ After converting the DrugBank identifiers to RxCUIs for databases with DDI severity information and excluding duplicates and symmetrical correspondences, 9868 DDIs were identified. Examples of specific data flow up to this point can be found in Figure S1.

**Figure 5.**
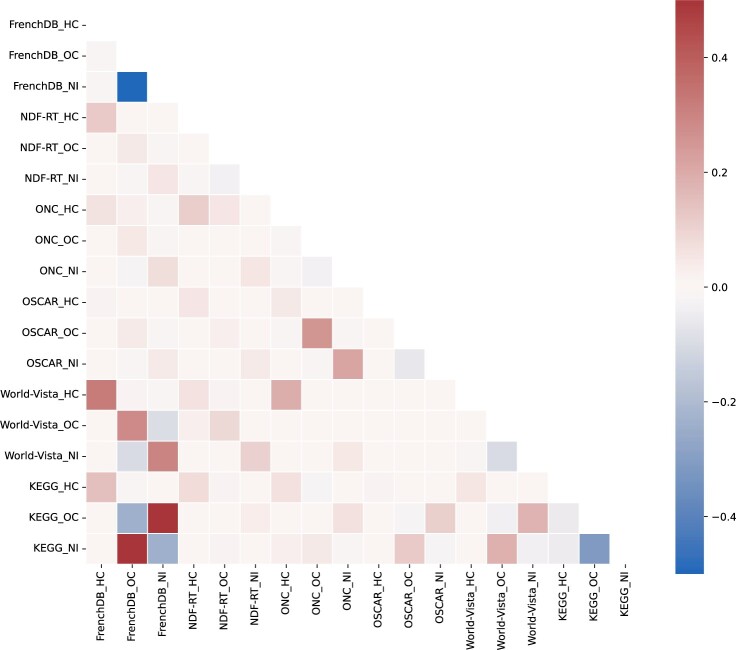
The extent of agreement among controlled drug-drug interaction severity classifications. Vertical and horizontal axes are categorized as the highest class (HC), other class (OC), and no information (NI). The values in the color bar represent the extent of agreement by Cohen’s kappa. 0.40-0.59 indicates weak agreement; 0.21-0.39 indicates minimal agreement; and 0.00-0.20 and below 0.00 indicate no agreement.

When integrating Japanese and international DDI information, we considered the DDI severity classifications of all sources; all drugs were paired with RxNorm concepts. We identified 36 947 DDIs. Figure 5 shows the correspondences among each source’s controlled DDI severity classifications; the heatmap was drawn after calculating Cohen’s kappa values (Supplementary Data). Overall, the DDI severity classification agreement was uniformly low across all sources, particularly in terms of the Japanese DDI information.

Table 3 shows the cross-tabulation results by the controlled DDI severity classification. Japanese and international DDI information is compared. The Japanese DDI information omitted 213 combinations of the highest international DDI class. Conversely, the international DDI information lacked 616 combinations that Japanese drug labels consider to be contraindicated. Examples of these combinations can be found in Tables S1 and S2.

**Table 3.** Cross-tabulation of the controlled DDI severity classifications.

		Japanese DDI severity information			Total
		Highest class	Other class	No information	
International DDI severity information	Highest class	149	285	213	647
	Other class	150	4194	4739	9083
	No information	616	26 471	130	27 217
	Total	915	30 950	5082	36 947

Abbreviation: DDI, drug-drug interaction.

## Discussion

### Relationship between YJ codes and RxNorm concepts

**Figure 6.**
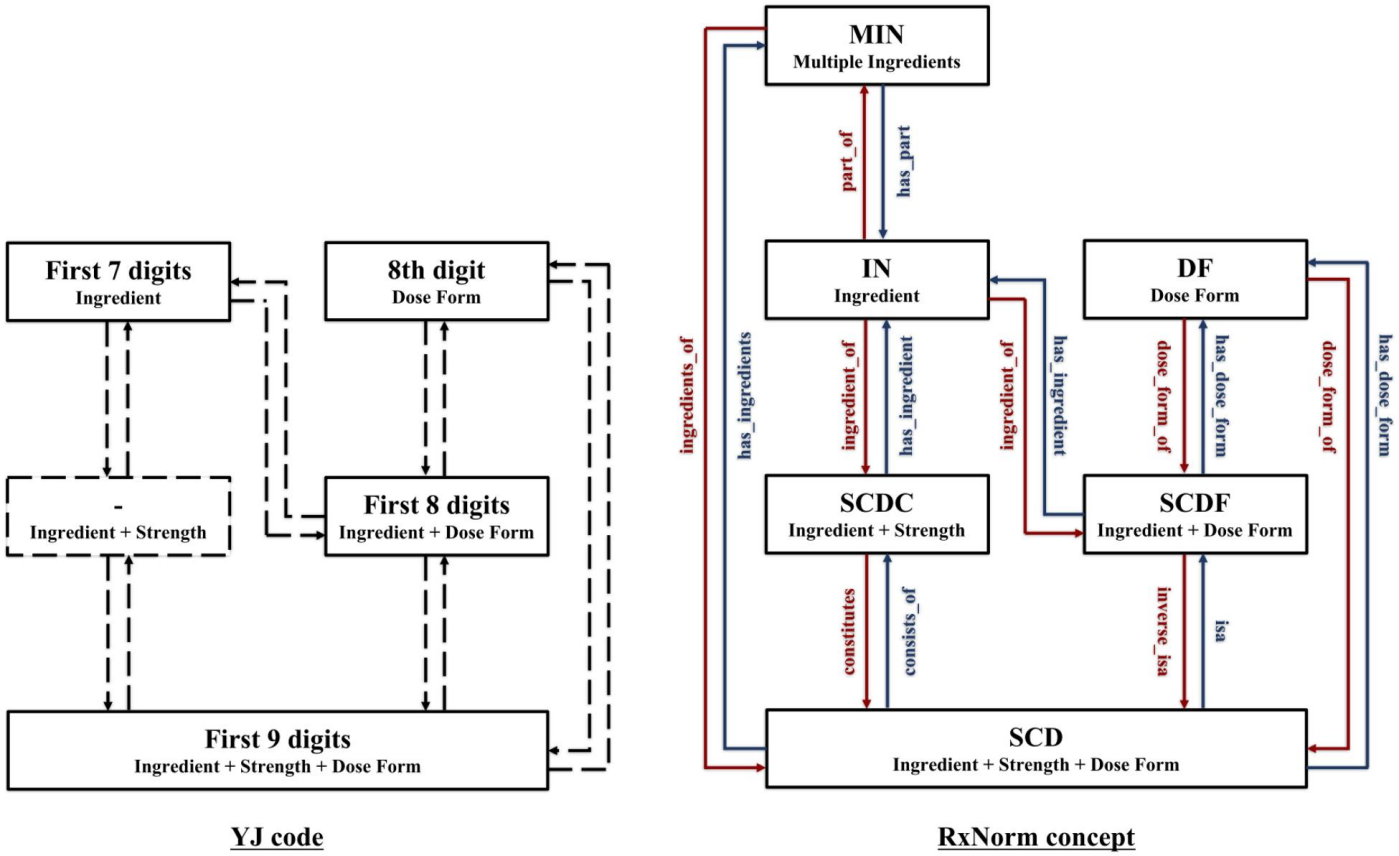
Correspondences within the network of YJ codes and RxNorm concepts.

Figure 6 illustrates correspondences between the network of YJ codes and RxNorm concepts. Of the latter, the Semantic Clinical Drug (SCD) class corresponds to the Ingredient (IN) and Dose Form (DF) classes, and their respective subordinate Semantic Clinical Drug Component (SCDC) and Semantic Clinical Drug Form (SCDF) classes. The Multiple Ingredients (MIN) class, which refers to drug ingredients, is also present. Strength is not defined as a class, but as an attribute of other classes. Our study used the IN or MIN class to compare Japanese and international DDI information.

The YJ code does not define class concepts in the manner of RxNorm. When applying the YJ code to the RxNorm classes, the first 9 digits of the YJ code (YJ9) are equivalent to the SCD class. The first 7 digits (YJ7) contain information analogous to that of the IN or MIN class. These data were considered adequately mapped drug ingredients. However, the first 7 digits of YJ codes may return both single and combination agents with identical ingredients; thus, the information does not uniquely identify ingredients. We determined that the unique 12-digit YJ code would be appropriate for linking to the IN or MIN class of RxNorm.

### Construction of the YJ-RxCUI table

The table of the correspondences between YJ codes and NDCs extracted from the KEGG DRUG database is based on the ingredient classes. The KEGG D numbers identify drug ingredients. However, no published mapping table that aligns these codes with the RxNorm concepts yet exists. International drug KBs frequently use RxNorm concepts or corresponding vocabularies. Mapping of YJ codes to RxNorm concepts is beneficial for leveraging international KB knowledge. We validated 416 (4%) correspondences between different ingredients of the YJ code and RxCUI in the YJ-RxCUI table. There are 2 principal causes by which correspondences with different ingredients may be recorded. First, for a certain drug, multiple ingredient correspondences are returned during conversion from NDC to RxCUI data using the RxNorm API. Although the RxNorm API parameters were adjusted to retrieve only the properties of active NDCs, some drugs retrieved inactive NDCs. For example, the NDC property “71335-1641” of the RxNorm API yields the RxCUIs for atorvastatin and gabapentin. A review of the NDC gabapentin history revealed that the NDC was inactive in September 2020. Consequently, a single NDC outputs multiple RxCUIs, generating correspondences with different ingredients. It has been suggested that conversion from NDC to RxCUI format using RxNorm API must exclude inactive correspondences in the NDC history. Future studies should incorporate such exclusions. The second issue involves disagreements between KEGG D numbers and NDCs with different ingredients in the KEGG DRUG database. For example, the ingredient associated with KEGG D number “D00630” is isosorbide mononitrate, which corresponds to the relevant NDC. However, KEGG D number “D00630” also corresponds to timolol of NDC “17478-189.” A substantial proportion of the correspondence among different ingredients in the KEGG DRUG database relates liquids used for dissolution and medical gases, which may differ between Japanese and internationally approved items. Therefore, various approved items and their international database annotations warrant attention. The KEGG DRUG database lacks information on dose form and strength. As identical ingredients may have different drug information if the dose forms or strengths vary, addressing these issues in more detailed studies are necessary.

The correspondence table facilitated the linkage of Japanese pharmaceutical products to KBs that employed RxNorm concepts or corresponding vocabularies. The correspondence table may show a one-to-many relationship between YJ codes and RxNorm IN classes; the YJ codes do not establish a hierarchical network of ingredients, dose forms, and strengths, which results from an inability to classify multi-ingredient drugs by their ingredients. The KEGG provides correspondence between the YJ codes and the Anatomical Therapeutic Chemical Classification System (ATC) codes.^26^ However, in terms of therapeutic effects, the ATC code does not uniquely identify ingredients and therefore does not contribute to mapping by ingredient class, which was the objective of the present study. In addition, the ATC code does not construct a hierarchical network of ingredients, dose forms, and strengths, precluding its use for mapping dose forms and strengths employing RxNorm concepts. Consequently, we revealed that no systematic, publicly available Japanese database has considered dose forms and strengths yet. In the future, it will be important to derive open data on dose forms and strengths that map to SCDs, including their ingredients.

As shown in Figure 6, the RxNorm concept has established a multifunctional hierarchical network. In this study, we constructed a correspondence table in which drugs with multiple ingredients were mapped to both the IN and MIN classes.

### Japanese and international DDI information

The Japanese drug labels of the KEGG MEDICUS DDI database use “CI: Contraindications” and “P: Precautions” to indicate DDI severity; sometimes, both CI and P are employed. For example, mirabegron, a selective β3-adrenergic receptor agonist used to treat overactive bladders, is contraindicated if a patient takes flecainide to treat tachyarrhythmia. However, flecainide is a “CYP2D6 substrate drug” tagged with “Precautions.” The severity of other DDIs also varies; for example, the DDI severity of the direct renin inhibitor aliskiren varies according to diabetes status. We did not consider clinical information; we studied only drug data. Therefore, we classified DDIs that included CI as the “Highest class,” and indicated only P as “Other class.” We also compared DDI information only for ingredient classes in this study. However, drug effects may differ in the way interactions appear depending on the route of administration. These are limitations of our study; future research on DDI severity must incorporate clinical information and the route of administration, to provide more detailed severity classifications. Furthermore, we tentatively classified the DDI severity to highlight the possibility of widely differing opinions on serious severity. In the future, it will be necessary to consider aligning classification criteria toward unifying international severity classifications.

A comparison of Japanese and international DDI information revealed that most precautions on Japanese drug labels correspond to “No information” in the international severity classification. Precautions on Japanese drug labels are sometimes not described as DDIs with specific drugs, but rather as DDIs with groups, such as therapeutic categories. For example, “diuretics,” “drugs that are substrates for CYP3A,” and “drugs known to cause QT prolongation” indicate DDIs with multiple drugs. The KEGG MEDICUS DDI database links such groups to drugs based on a unique list. For some groups (eg, “drugs that are substrates for CYP3A”), a very large number of drugs are listed. Consequently, most of the “Precautions” in the Japanese DDI information were classified as “No information” in the international DDI information.

The low concordance between Japanese and international DDI severity levels suggests that clinically essential data should be added to the Japanese and international DDI information. This means there are no clear internationally agreed-upon criteria for setting DDI severity, and severity information on DDI needs to be coordinated to be consistent. Note that DDI systems based solely on Japanese or international information are at risk of lacking information on serious DDIs. Normalizing all DDI data sources to the RxNorm concept and linking them to the international KB may efficiently identify DDIs that should be reviewed by checking for differences regarding severity.

## Conclusion

We mapped the YJ codes to ingredient classes of RxNorm concepts and facilitated the linkage of Japanese pharmaceutical products to an international KB. Our focus was on ingredient classes; in a future study, we will derive mapping methods that incorporate dose forms and strengths. We verified that international knowledge could be leveraged by integrating and comparing Japanese and international DDI information.

## Supplementary Material

ocae094_Supplementary_Data

## Data Availability

The data that support this study are derived from the KEGG and NLM. Redistribution of such data is prohibited.
