# Supplementary material for: Toward a unified understanding of drug-drug interactions: mapping Japanese drug codes to RxNorm concepts
Source: J Am Med Inform Assoc. 2024 May 17;31(7):1561–8. doi: 10.1093/jamia/ocae094 (PMC11187495; doi:10.1093/jamia/ocae094)
Supplement: ocae094_Supplementary_Data [file ocae094_supplementary_data.zip › ocae094_Supplementary_Data/SupplementaryFigure.pdf]

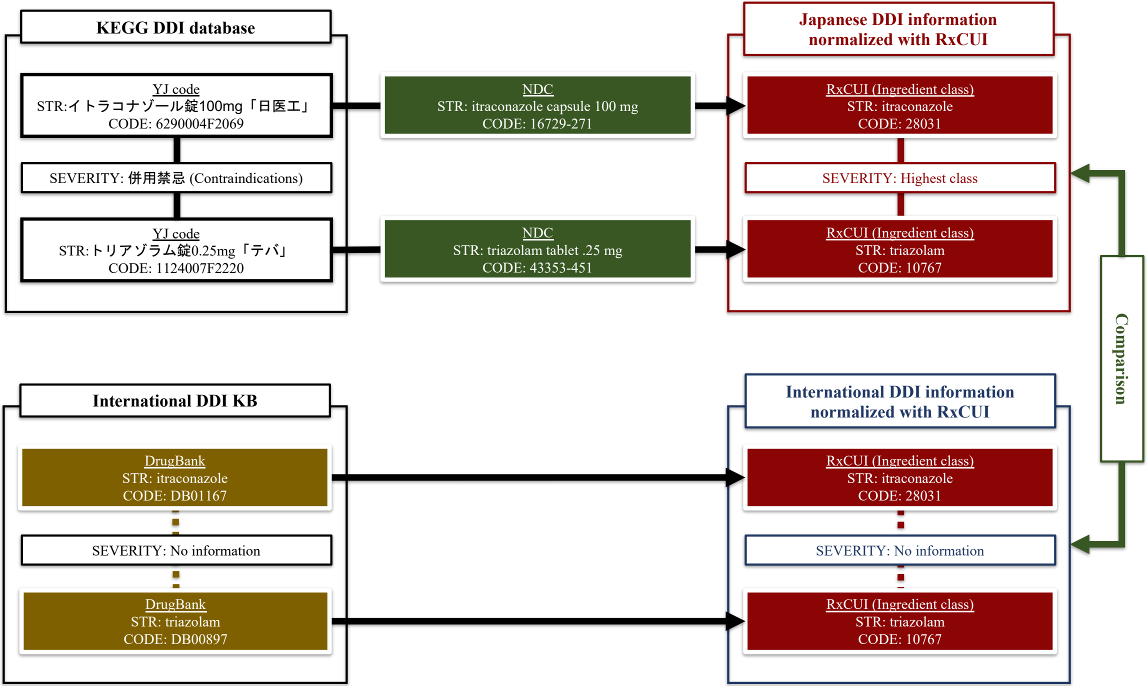

Supplementary Figure. An example of correspondence of drug codes and DDIs.

* DDI: Drug-Drug Interaction; KB: Knowledge Base; STR: String; NDC: National Drug Code; RxCUI: RxNorm Concept Unique Identifier.
