# Supplementary material for: Toward a unified understanding of drug-drug interactions: mapping Japanese drug codes to RxNorm concepts
Source: J Am Med Inform Assoc. 2024 May 17;31(7):1561–8. doi: 10.1093/jamia/ocae094 (PMC11187495; doi:10.1093/jamia/ocae094)
Supplement: ocae094_Supplementary_Data [file ocae094_supplementary_data.zip › ocae094_Supplementary_Data/SupplementaryTable.docx]

**SUPPLEMENTARY MATERIAL**

Supplementary Table 1. An example of a combination of DDIs
that “No information” in Japanese and “Highest class” in international.

| RxCUI1 | Ingredient1 | RxCUI2 | Ingredient2 | Japanese  DDI severity  information | International  DDI severity  information |
| --- | --- | --- | --- | --- | --- |
| 2403 | chlorpromazine | 703 | amiodarone | NI | HC |
| 3648 | droperidol | 2403 | chlorpromazine | NI | HC |
| 60307 | entacapone | 190376 | linezolid | NI | HC |
| 190376 | linezolid | 10737 | trazodone | NI | HC |
| 26225 | ondansetron | 3648 | droperidol | NI | HC |
| 322167 | solifenacin | 85762 | ritonavir | NI | HC |
| 9947 | sotalol | 4053 | erythromycin | NI | HC |
| 57258 | tizanidine | 281 | acyclovir | NI | HC |
| 57258 | tizanidine | 4278 | famotidine | NI | HC |
| 121243 | voriconazole | 703 | amiodarone | NI | HC |

* RxCUI1 (Ingredient1) and RxCUI2 (Ingredient2) are the unique identifier and ingredient of the RxNorm concept that is the pair of DDIs, respectively.

* DDI: Drug–Drug Interaction; RxCUI: RxNorm Concept Unique Identifier; HC: Highest class; NI: No information.

Supplementary Table 2. An example of a combination of DDIs
that “Highest class” in Japanese and “No information” in international.

| RxCUI1 | Ingredient1 | RxCUI2 | Ingredient2 | Japanese  DDI severity  information | International  DDI severity  information |
| --- | --- | --- | --- | --- | --- |
| 296 | adenosine | 3521 | dipyridamole | HC | NI |
| 343047 | atazanavir | 1114195 | rivaroxaban | HC | NI |
| 21212 | clarithromycin | 1547099 | suvorexant | HC | NI |
| 2582 | clindamycin | 4053 | erythromycin | HC | NI |
| 4450 | fluconazole | 1040028 | lurasidone | HC | NI |
| 28031 | itraconazole | 10767 | triazolam | HC | NI |
| 6932 | miconazole | 1364479 | lomitapide | HC | NI |
| 42316 | tacrolimus | 75207 | bosentan | HC | NI |
| 38413 | torsemide | 3251 | desmopressin | HC | NI |
| 11413 | zidovudine | 5640 | ibuprofen | HC | NI |

* RxCUI1 (Ingredient1) and RxCUI2 (Ingredient2) are the unique identifier and ingredient of the RxNorm concept that is the pair of DDIs, respectively.

* DDI: Drug–Drug Interaction; RxCUI: RxNorm Concept Unique Identifier; HC: Highest class; NI: No information.
